# Impact of Concomitant Meniscus Repair With Anterior Cruciate Ligament Reconstruction on Functional Outcomes at Short-Term Follow-Up: A Prospective Observational Study

**DOI:** 10.7759/cureus.113786

**Published:** 2026-08-01

**Authors:** Pratyaksh K Gupta, Kailash Jaidev, Amit Mahajan, Rohit Kulkarni, Snehal Hedgire, Nishant Mirchandani

**Affiliations:** 1 Department of Orthopaedic Surgery, Bharati Vidyapeeth (Deemed to be University) Medical College, Pune, IND; 2 Department of Orthopaedics and Trauma, Bharati Hospital, Pune, IND; 3 Department of Orthopaedics, Bharati Vidyapeeth (Deemed to be University) Medical College, Pune, IND

**Keywords:** acl surgery, arthroscopy, meniscus surgery, sports and arthroscopy, sports rehabilitation

## Abstract

Background: Anterior cruciate ligament (ACL) ruptures are among the most common knee injuries and impose a substantial health burden. ACL injuries are common, and meniscal tears frequently co-occur with acute ACL ruptures, most often involving the medial meniscus. Proper management of combined ACL and meniscus injuries is critical to restore knee function and prevent long-term degeneration. Although concurrent meniscal repair with ACL reconstruction (ACLR) is increasingly favoured over meniscectomy, reported patient-reported outcomes remain inconsistent across registries and are drawn largely from retrospective, Western cohorts, leaving uncertainty about short-term recovery in prospectively followed South Asian populations.

Methods: We conducted a single-centre prospective observational study of 115 consecutive patients (mean age = 31.3 ± 10.5 years; range = 17-58) undergoing arthroscopic ACLR with concurrent meniscal repair. All patients were assessed preoperatively and postoperatively at six weeks, three months, and six months using the visual analogue scale (VAS) for pain, the Lysholm Knee Score, and the International Knee Documentation Committee (IKDC) subjective score. Because the Shapiro-Wilk test showed all outcome variables were non-normally distributed at every time point (p < 0.05), non-parametric methods were used: the Friedman test assessed overall change across the four time points, and Wilcoxon signed-rank tests (Bonferroni-corrected) compared each postoperative time point with baseline. Data are summarised as median (interquartile range, IQR); effect sizes are Kendall's W (Friedman) and r (Wilcoxon). A two-tailed p < 0.05 was considered significant.

Results: Of 115 patients, 92 (80.0%) were male, and 23 (20.0%) were female; medial meniscus tears were present in 61 (53.0%), lateral in 39 (33.9%), and combined medial-lateral in 15 (13.0%). All 115 patients completed six-month follow-up with no losses to follow-up, and no intraoperative or postoperative complications (infection, graft failure, or re-tear) were observed. VAS pain improved from a median of 4.0 (IQR = 3.0-5.0) preoperatively to 0.0 (0.0-0.0) at six months (Friedman χ²(3) = 298.7, p < 0.001; Wilcoxon vs. preoperatively p < 0.001, r = 0.87). The Lysholm score improved from 66.0 (60.0-73.0) to 94.0 (94.0-94.0) (Friedman χ²(3) = 292.3, p < 0.001; r = 0.86 at six months). The IKDC subjective score transiently declined at six weeks (median = 42.5 vs. 44.8 preoperatively, p < 0.001) before improving to 77.0 (72.4-81.6) at six months (Friedman χ²(3) = 278.1, p < 0.001; r = 0.87). All pairwise comparisons against baseline were significant except the Lysholm score at six weeks (p ≈ 0.05).

Conclusion: In this single-arm prospective cohort, arthroscopic ACLR with concurrent meniscal repair was associated with significant improvements in pain and patient-reported knee function over six months, with no observed complications. Because the study lacked a comparison group, these findings demonstrate favourable within-cohort outcomes and suggest that incorporating meniscal repair into ACLR does not appear to compromise short-term recovery; they cannot establish the efficacy or comparative effectiveness of the intervention. Further comparative and longitudinal studies are warranted.

## Introduction

Anterior cruciate ligament (ACL) injuries are among the most prevalent and debilitating knee injuries in athletic populations and impose a substantial health burden [1,2]. Reported incidence figures reach approximately 68 per 100,000 person-years, with the highest rates in young, pivoting-sport athletes [2]. Meniscal tears are commonly associated with ACL ruptures, co-occurring in an estimated 50-75% of cases, especially the medial meniscus, which is more tightly anchored and thus prone to injury during episodes of instability [3]. Concomitant meniscal injury can exacerbate knee instability and, if untreated, accelerate cartilage wear leading to early osteoarthritis. Historically, meniscus tears underwent meniscectomy, and this led to an increased risk of joint degeneration in the long term [4]. Therefore, preserving and repairing the meniscus whenever possible is crucial for maintaining knee biomechanics and health.

Arthroscopic ACL reconstruction (ACLR) combined with meniscal repair has become a standard approach for knees with concurrent ACL and meniscus tears. Several studies have indicated that performing meniscal repair at the time of ACLR yields favourable outcomes, with improvements in knee stability and patient-reported function [5].

However, the evidence base remains inconsistent, and important gaps persist. Large registry analyses report conflicting results: LaPrade and colleagues, using the Norwegian Knee Ligament Registry, found no significant two-year difference in patient-reported outcomes between patients undergoing concurrent meniscal surgery and those receiving isolated ACLR, whereas Svantesson et al. reported inferior one-year symptom and activity scores after concomitant medial meniscal repair, and a recent meta-analysis found no clinically significant short-term difference between repair and meniscectomy [6-8]. These divergent conclusions are further limited by the predominance of retrospective designs, heterogeneous rehabilitation protocols, variable follow-up durations, and the concentration of data in North American and European registries. Prospective data from South Asian populations, where patient demographics, activity profiles, and access to structured rehabilitation may differ, are scarce. This uncertainty, whether the favourable results reported elsewhere are reproduced when meniscal repair is added to ACLR, and whether patients are followed prospectively, provided the rationale for the present study.

Against this background, the present study aimed to prospectively evaluate the changes in pain and patient-reported knee function following arthroscopic ACLR with concurrent meniscal repair over a six-month follow-up in a single South Asian tertiary-care cohort. The specific objectives were (1) to quantify changes in the visual analogue scale (VAS) pain score [9], the Lysholm Knee Score [10], and the International Knee Documentation Committee (IKDC) subjective score [11] from the preoperative baseline to six weeks, three months, and six months postoperatively, and (2) to document the intra- and postoperative complication rate. We hypothesised that patients would show significant improvements in knee function and pain relief by six months postoperatively, without a high complication rate. Consistent with the observational single-arm design, this study was designed to describe within-cohort change over time rather than to establish the efficacy of the intervention relative to any comparator.

## Materials and methods

Study design, participants, and recruitment

This was a single-centre prospective observational cohort study conducted at a tertiary-care centre. As a single-group observational study, it followed one cohort over time without a comparison or control arm; no allocation to alternative treatments was performed. Ethical approval was obtained from the institutional ethics committee prior to commencement (BVDUMC/IEC/125), and the study was prospectively registered with the Clinical Trials Registry of India (CTRI/2023/08/056400). Written informed consent was obtained from all participants. Consecutive eligible patients presenting to the orthopaedic outpatient and emergency services who were scheduled for arthroscopic ACLR with concurrent meniscal repair were recruited between June 2023 and August 2024 (consecutive sampling). Inclusion criteria were patients aged 16-65 years with an ACL tear and a reparable meniscal tear (medial, lateral, or both) confirmed on MRI who consented to combined ACL reconstruction and meniscal repair. Exclusion criteria were multi-ligament knee injuries, partial ACL tears not requiring reconstruction, advanced pre-existing knee osteoarthritis (Kellgren-Lawrence grade 3 or 4), or isolated ACL injuries without meniscal involvement. A formal a priori sample-size calculation was not performed; instead, all consecutive eligible patients presenting during the 15-month enrolment window were included, yielding a convenience sample of 115 patients. A post-hoc assessment confirmed that this sample provided greater than 99% power to detect the observed within-subject changes at a two-sided α of 0.05; the absence of a formal a priori calculation is acknowledged as a limitation. All 115 enrolled patients completed the full six-month follow-up, with no dropouts or losses to follow-up.

Outcome measures

Baseline demographic and injury data were recorded, including age, sex, comorbidities, meniscal tear location, and the interval from initial injury to surgery. Clinical knee examination (Lachman and anterior drawer tests) confirmed ACL insufficiency in all patients preoperatively. The outcome measures were three validated patient-reported instruments, selected because they capture complementary domains of the knee-injury experience, i.e., pain, activity-related symptoms, and global subjective function, and are the most widely used measures in ACL and meniscal outcome research, facilitating comparison with prior literature. The VAS for pain (0 = no pain, 10 = worst pain) is a simple, responsive measure of pain intensity with established reliability and validity [9]. The Lysholm Knee Score (0-100; higher scores indicate better function and stability) is a validated, reliable knee-specific instrument [10]. The IKDC subjective knee evaluation form (0-100) is a validated and reliable region-specific patient-reported outcome for knee disorders, including ACL injury [11]. Each instrument was administered by a trained assessor at four time points, i.e., preoperatively and at six weeks, three months, and six months postoperatively, and the same instruments were used at every visit so that preoperative and each postoperative value could be directly compared within each patient. The primary endpoints were the within-patient changes in VAS, Lysholm, and IKDC scores from the preoperative baseline to each postoperative time point. Any intra- or postoperative complications were documented. No routine imaging or second-look arthroscopy was performed to confirm meniscal healing.

Rehabilitation protocol

All patients received the same surgical intervention, i.e., arthroscopic ACL reconstruction with concurrent meniscal repair, followed by an identical, standardised postoperative rehabilitation protocol supervised by qualified physiotherapists at the treating institution; no unsupervised (technician-, aide-, or family-administered) rehabilitation substituted for the supervised programme. For the initial six weeks, knee range of motion was restricted to protect the meniscal repair, and patients were kept non-weight-bearing with crutches for the first four to six weeks to avoid excessive load on the healing meniscus. During this period, closed kinetic chain exercises, including quadriceps activation, straight-leg raises, and gentle range-of-motion work within permitted limits, were performed to prevent stiffness. From six to 12 weeks, range of motion was gradually progressed to full as tolerated, and strengthening (including open kinetic chain) exercises emphasising both quadriceps and hamstrings were introduced; neuromuscular and proprioceptive training was incorporated as weight-bearing increased. After three months, more advanced strengthening and functional training were permitted. Return to full sport was guided by established, criterion-based return-to-sport benchmarks, including restoration of near-symmetrical quadriceps and hamstring strength, full painless range of motion, absence of effusion, and completion of a sport-specific functional progression, rather than by time alone, and was generally not before six months, on a case-by-case basis [12]. Adherence to the supervised programme was reinforced and reviewed at each scheduled follow-up visit.

Statistical analysis

All analyses were performed using IBM SPSS Statistics for Windows, version 29.0 (IBM Corp., Armonk, NY). The distribution of each continuous outcome (VAS, Lysholm, and IKDC scores) at every time point was assessed for normality using the Shapiro-Wilk test, supported by visual inspection of histograms and Q-Q plots. All variables were non-normally distributed at all time points (Shapiro-Wilk p < 0.05); accordingly, non-parametric methods were used, and continuous variables are summarised as median (interquartile range, IQR) in addition to mean ± standard deviation. Categorical variables (sex, meniscal tear location, comorbidities) are expressed as frequencies and percentages. Because the outcomes were measured repeatedly in the same patients across four time points, the Friedman test (the non-parametric analogue of repeated-measures ANOVA, which does not require the assumptions of normality or sphericity) was used to assess overall change over time for each score. Where the Friedman test was significant, pre-planned pairwise comparisons between the preoperative value and each postoperative time point were performed using the Wilcoxon signed-rank test, with a Bonferroni correction for the three comparisons per outcome. Effect sizes are reported as Kendall's W for the Friedman test and as r (Z/√N) for Wilcoxon comparisons. A two-tailed p < 0.05 (after Bonferroni adjustment, where applicable) was considered statistically significant. The originally planned repeated-measures ANOVA and paired t-tests were not used because the normality assumption underpinning those parametric tests was violated for every variable.

## Results

**Figure 1:**
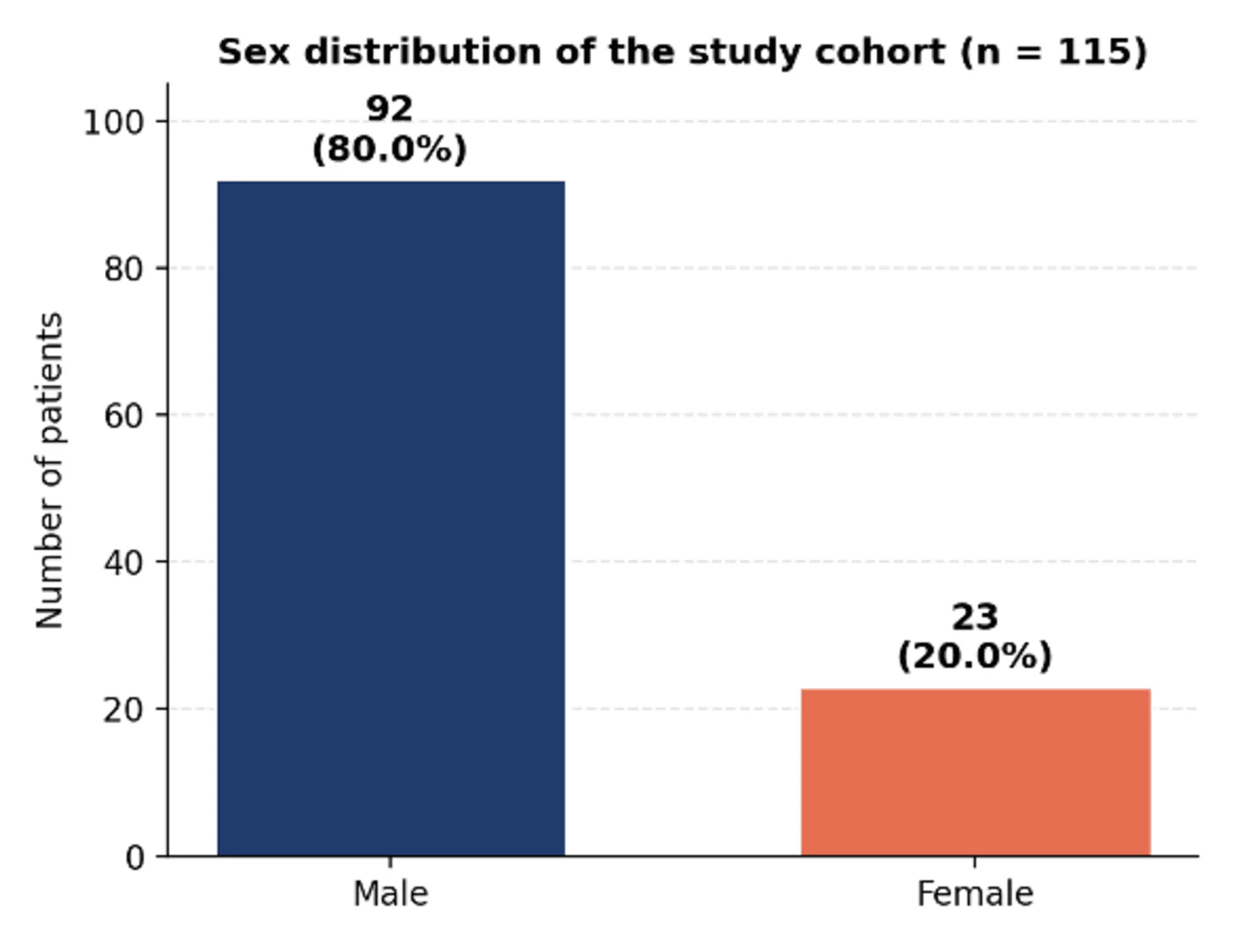
Sex distribution of the study cohort (n = 115). Of the 115 patients who underwent arthroscopic anterior cruciate ligament reconstruction with concurrent meniscal repair, 92 (80.0%) were male and 23 (20.0%) were female.

A total of 115 patients (92 (80.0%) male; mean age = 31.3 ± 10.5 years, range = 17-58) were enrolled, and all completed the six-month follow-up, with no dropouts or losses to follow-up (Figure 1). The median interval from initial injury to surgery was 2.1 months (IQR = 1.0-3.4). Meniscal tear distribution was 61 medial (53.0%), 39 lateral (33.9%), and 15 combined (13.0%). Comorbidities were uncommon, including hypertension in five (4.3%) and diabetes mellitus in one (0.9%). Baseline demographic and clinical characteristics are summarised in Table 1. No intraoperative or postoperative complications occurred, including graft or meniscal-repair failures.

**Table 1:** Baseline demographic and clinical characteristics of the study cohort (n = 115). SD, standard deviation; IQR, interquartile range. Body mass index, graft type, and detailed meniscal tear morphology (zone/pattern) were not routinely recorded and are acknowledged as a limitation.

Characteristic	Value (n = 115)
Age, years — mean ± SD (range)	31.3 ± 10.5 (17-58)
Sex — male, n (%)	92 (80.0)
Sex — female, n (%)	23 (20.0)
Time from injury to surgery, months — median (IQR)	2.1 (1.0-3.4)
Meniscal tear — medial, n (%)	61 (53.0)
Meniscal tear — lateral, n (%)	39 (33.9)
Meniscal tear — combined medial + lateral, n (%)	15 (13.0)
Comorbidity — hypertension, n (%)	5 (4.3)
Comorbidity — diabetes mellitus, n (%)	1 (0.9)
Comorbidity — none, n (%)	109 (94.8)

**Figure 2:**
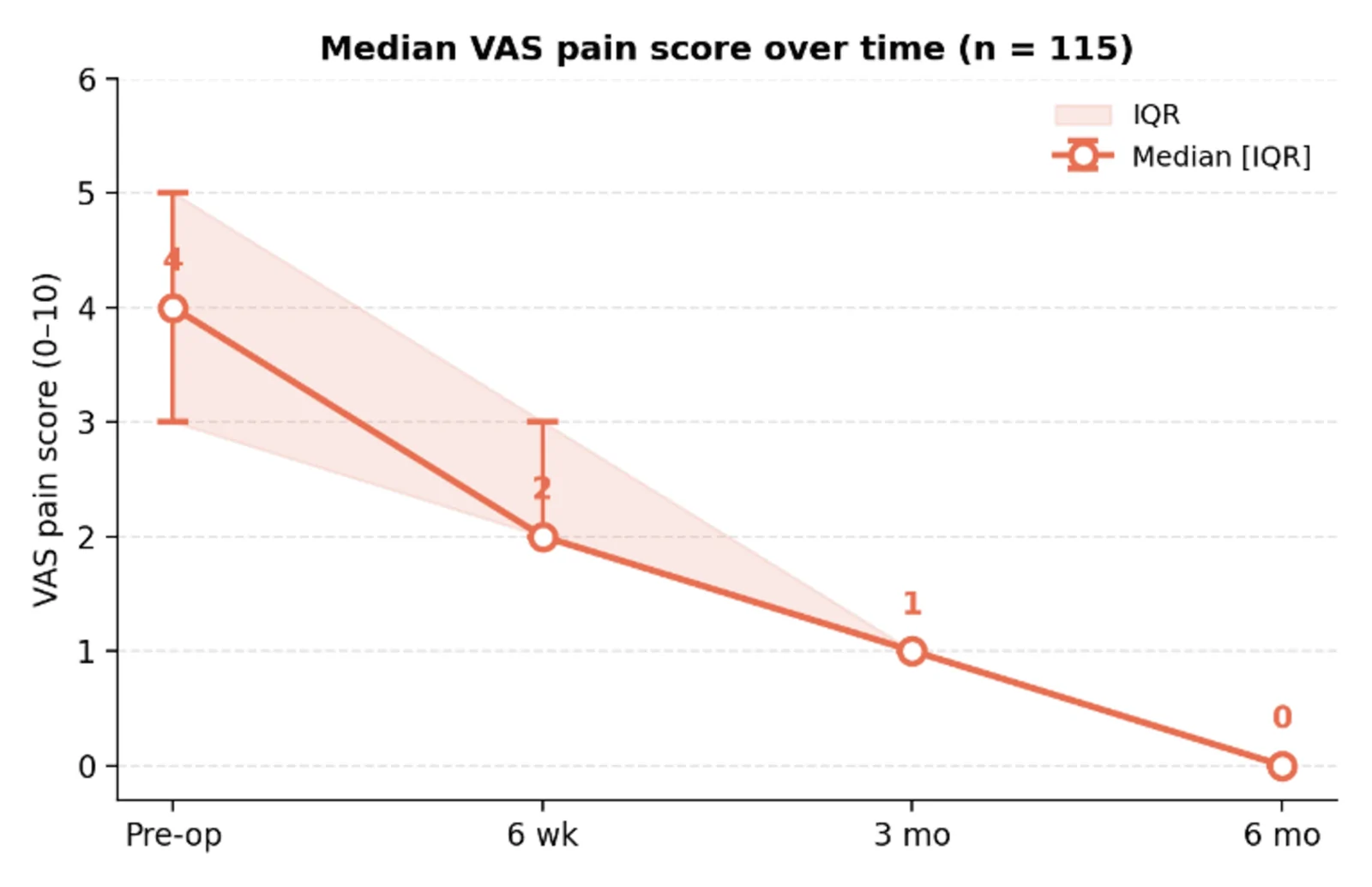
Median visual analogue scale (VAS) pain score with interquartile range (IQR) from the preoperative visit through six months postoperatively (n = 115). Pain decreased from a median of 4.0 (IQR = 3.0-5.0) preoperatively to 0.0 (0.0-0.0) at six months (Friedman χ²(3) = 298.7, p < 0.001; Wilcoxon vs. preoperative p < 0.001 at every time point).

**Figure 3:**
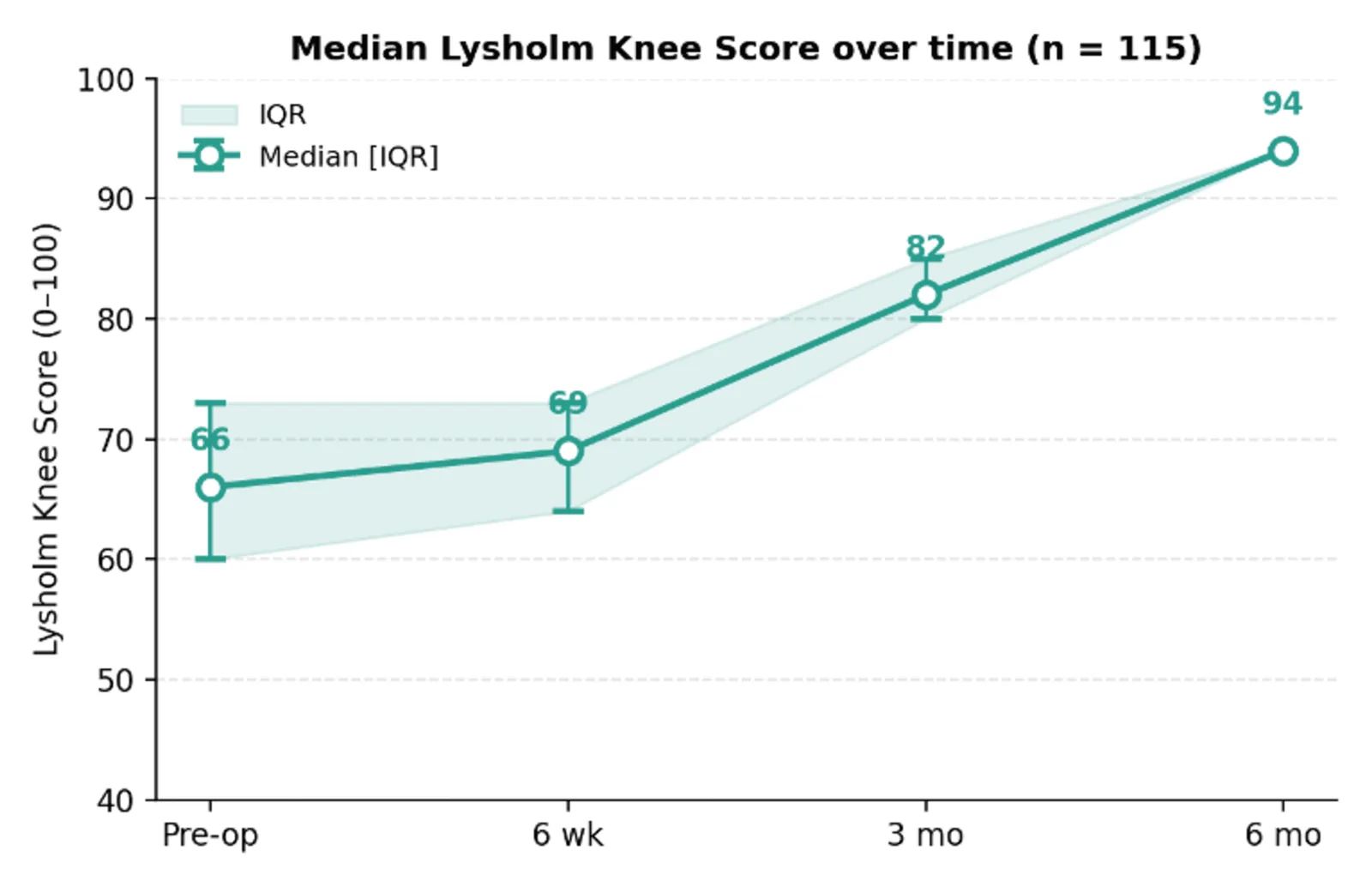
Median Lysholm Knee Score with interquartile range (IQR) from the preoperative visit through six months postoperatively (n = 115). The median improved from 66.0 (60.0-73.0) preoperatively to 69.0 at six weeks, 82.0 at three months, and 94.0 at six months (Friedman χ²(3) = 292.3, p < 0.001). The change at six weeks was not statistically significant (p ≈ 0.05).

**Figure 4:**
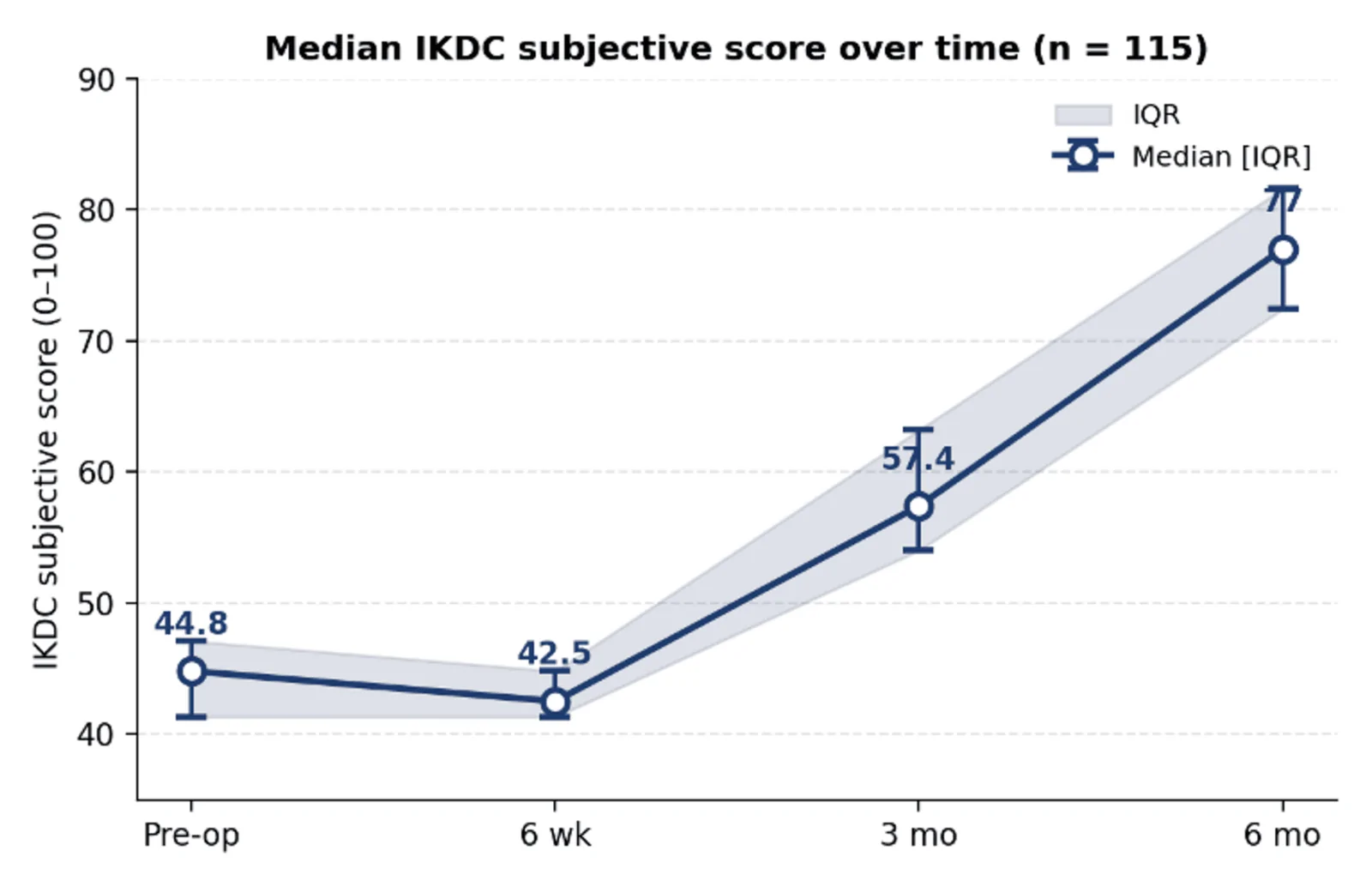
Median International Knee Documentation Committee (IKDC) subjective score with interquartile range (IQR) from the preoperative visit through six months postoperatively (n = 115). The score transiently declined at six weeks (median = 42.5 vs. 44.8 preoperatively), reflecting early postoperative pain and rehabilitation restrictions, before improving to 57.4 at three months and 77.0 at six months (Friedman χ²(3) = 278.1, p < 0.001), surpassing the preoperative baseline.

Because all outcome variables were non-normally distributed (Shapiro-Wilk p < 0.05 at every time point), results are reported as median (IQR) and analysed with the Friedman and Wilcoxon signed-rank tests (Tables 2-4). The Friedman test demonstrated a significant overall change over time for every outcome (VAS χ²(3) = 298.7; Lysholm χ²(3) = 292.3; IKDC χ²(3) = 278.1; all p < 0.001), with large effect sizes (Kendall's W = 0.81-0.87). VAS pain decreased from a median of 4.0 (IQR = 3.0-5.0) preoperatively to 0.0 (0.0-0.0) at six months, with significant reductions at every postoperative time point versus baseline (all p < 0.001; r = 0.72-0.87). The Lysholm score improved from 66.0 (60.0-73.0) to 94.0 (94.0-94.0); the improvement was significant at three and six months (both p < 0.001) but not significant at six weeks (p ≈ 0.05, r = 0.22), consistent with the early motion- and weight-bearing restrictions of the rehabilitation protocol. The IKDC subjective score transiently declined at six weeks (median = 42.5 vs. 44.8 preoperatively; p < 0.001) before improving to 57.4 (54.0-63.2) at three months and 77.0 (72.4-81.6) at six months (both p < 0.001), surpassing baseline. Tables 2-4 and Figures 2-4 summarise the progression of each score over time. Overall, arthroscopic ACLR with concurrent meniscal repair was associated with significant within-cohort improvements in pain and patient-reported knee function over the six-month period.

**Table 2:** Visual analogue scale (VAS) pain scores over time (0-10): preoperatively and at six weeks, three months, and six months postoperatively (n = 115). SD, standard deviation; IQR, interquartile range. * Wilcoxon signed-rank test versus preoperative value, Bonferroni-corrected for three comparisons. Overall Friedman test across all four time points: χ²(3) = 298.7, p < 0.001, and Kendall's W = 0.87.

Time point	Median (IQR)	Mean ± SD	Effect size r (vs. pre-op)	p (vs. pre-op)*
Preoperative	4.0 (3.0-5.0)	4.23 ± 1.69	—	—
6 weeks	2.0 (2.0-3.0)	2.45 ± 1.03	0.72	<0.001
3 months	1.0 (1.0-1.0)	1.10 ± 0.84	0.85	<0.001
6 months	0.0 (0.0-0.0)	0.03 ± 0.18	0.87	<0.001

**Table 3:** Lysholm Knee Score over time (0-100): preoperatively and at six weeks, three months, and six months postoperatively (n = 115). SD, standard deviation; IQR, interquartile range; NS, not significant after correction. * Wilcoxon signed-rank test versus preoperative value, Bonferroni-corrected. Overall Friedman test: χ²(3) = 292.3, p < 0.001, and Kendall's W = 0.85.

Time point	Median (IQR)	Mean ± SD	Effect size r (vs. pre-op)	p (vs. pre-op)*
Preoperative	66.0 (60.0-73.0)	65.80 ± 9.92	—	—
6 weeks	69.0 (64.0-73.0)	68.35 ± 3.78	0.22	0.050 (NS)
3 months	82.0 (80.0-85.0)	81.41 ± 3.26	0.83	<0.001
6 months	94.0 (94.0-94.0)	94.43 ± 2.27	0.86	<0.001

**Table 4:** International Knee Documentation Committee (IKDC) subjective score over time (0-100): preoperatively and at six weeks, three months, and six months postoperatively (n = 115). SD, standard deviation; IQR, interquartile range. * Wilcoxon signed-rank test versus preoperative value, Bonferroni-corrected. The reduction at six weeks reflects early postoperative pain and rehabilitation restrictions. Overall Friedman test: χ²(3) = 278.1, p < 0.001, and Kendall's W = 0.81.

Time point	Median (IQR)	Mean ± SD	Effect size r (vs pre-op)	p (vs. pre-op)*
Preoperative	44.8 (41.3–47.1)	47.87 ± 11.13	—	—
6 weeks	42.5 (41.3–44.8)	42.73 ± 2.98	0.34	<0.001
3 months	57.4 (54.0–63.2)	57.87 ± 5.40	0.72	<0.001
6 months	77.0 (72.4–81.6)	77.14 ± 6.00	0.87	<0.001

Figures 1-4 were regenerated directly from the study data to reflect the non-parametric analysis; the trend figures now display the median with the IQR at each time point.

## Discussion

Meniscal repair concurrent with ACLR has become increasingly accepted because it preserves joint biomechanics and may help prevent early-onset osteoarthritis. Historically, meniscectomy was the standard treatment for tears; however, it is now associated with increased cartilage degradation and accelerated joint degeneration and, in athletes, with a shortened playing career [13], making repair in vascular zones (red-red/red-white) the preferred approach when feasible [5,14,15].

Modern arthroscopic techniques, especially all-inside repair systems, have simplified meniscal repair and reduced the complications associated with inside-out or outside-in methods while providing stable fixation of peripheral tears [5,16,17]. Concomitant ACLR offers biological advantages, i.e., marrow-derived cytokines and an enhanced vascular response promote meniscal healing, which has been associated with high success rates in meta-analyses of repairs performed with ACLR [1,18]. Meniscus repair coupled with ACLR has also been associated with a lower rate of reoperations in some studies [19].

Our findings should be interpreted in the context of an inconsistent literature. LaPrade et al. examined 4,691 primary ACLR patients from the Norwegian Ligament Registry; preoperative Knee Injury and Osteoarthritis Outcome Score (KOOS) ratings were lower in patients with a concurrent medial or lateral meniscus tear, but two-year postoperative KOOS did not differ significantly between patients who underwent concurrent meniscal surgery and those who received ACLR alone [6]. In a retrospective cohort of 6,398 primary ACLR patients, Svantesson et al. found that patients undergoing concomitant medial meniscal repair had lower KOOS symptom and activities in daily living (ADL) scores at one-year follow-up [7]. A recent meta-analysis comparing meniscal repair with meniscectomy during primary ACLR found no clinically significant difference in patient-reported outcomes at short-term follow-up [8], underscoring that reported outcomes vary with registry, follow-up duration, and comparator group. Pathak et al.'s retrospective series demonstrated marked functional improvement and a low meniscal re-tear rate after combined ACLR and meniscal repair over mid-term follow-up [16]. Within this mixed evidence base, our prospective single-arm cohort adds short-term data from a South Asian population and shows favourable functional gains and pain relief at six months with no graft- or repair-related complications. These within-cohort results are consistent with the favourable early outcomes reported for combined procedures [1,16] and with the view that meniscal preservation alongside ACLR does not appear to compromise short-term recovery [16,20]. Because our study had no comparison group, however, it cannot determine whether these outcomes are superior, equivalent, or inferior to isolated ACLR, meniscectomy, or the natural course of recovery.

The transient decline in the IKDC score at six weeks, and the absence of a significant Lysholm change at the same time point, are consistent with the protective early rehabilitation phase; restricted weight-bearing and controlled range of motion protect the repair, while the subsequent introduction of strengthening and neuromuscular training coincided with the marked gains observed at three and six months. This pattern parallels rehabilitation protocols described by Westermann et al. and others [5,19], and, when combined with precise graft placement and tunnel positioning as emphasised by Rahardja et al., the risk of re-tear is reduced [21].

The choice of VAS, Lysholm, and IKDC as outcome measures reflects their status as validated, responsive, and widely reported instruments for pain and knee-specific function, which allows our results to be compared with the existing ACL and meniscal literature [9-11]. Nonetheless, these are patient-reported measures; they were not supplemented by objective performance testing, which is a limitation discussed below.

Limitations

Several limitations should be acknowledged. First, this was a single-arm observational cohort without a comparison or control group; consequently, the observed improvements cannot be attributed causally to the intervention and cannot be compared with alternative treatments (isolated ACLR or meniscectomy) or with the natural course of recovery, and no claim of efficacy or comparative effectiveness can be made. Second, the cohort was recruited from a single tertiary-care centre in South Asia and was predominantly male (80%), which limits the generalisability of the findings to female patients and to other geographic regions, healthcare systems, and patient populations. Third, outcomes were assessed exclusively with patient-reported instruments (VAS, Lysholm, IKDC); objective functional performance tests (e.g., single-leg hop tests and isokinetic strength testing) and radiographic or second-look arthroscopic confirmation of meniscal healing were not performed, limiting conclusions about true functional recovery and structural healing. Fourth, follow-up was limited to six months, precluding assessment of repair durability, re-tear, and long-term osteoarthritis risk. Fifth, a formal a priori sample-size calculation was not performed, and additional participant characteristics that may influence outcomes, including body mass index, preinjury activity level, graft type, and detailed meniscal tear morphology, were not routinely recorded, so their potential confounding effect could not be assessed. Finally, although all 115 patients completed follow-up with no attrition and adherence to the supervised rehabilitation programme was reinforced at each visit, adherence was not formally quantified. Future research should include prospective comparative designs with control groups, objective functional and imaging endpoints, longer follow-up, multi-centre and sex-balanced recruitment, and formal quantification of rehabilitation adherence to build on the present findings.

## Conclusions

In this single-arm prospective observational cohort, arthroscopic ACLR combined with meniscal repair was associated with significant improvements in pain and patient-reported knee function, across the VAS, Lysholm, and IKDC scores, over six months, with no observed intra- or postoperative complications. Because the study lacked a comparison group and relied on patient-reported outcomes over a short follow-up, these results demonstrate favourable within-cohort recovery and suggest that incorporating meniscal repair into ACLR does not appear to compromise short-term recovery; they should not be interpreted as establishing the efficacy or superiority of the combined procedure over alternative treatments. These findings are consistent with the biological rationale for meniscal preservation and with prior reports, and they support meniscal repair as a reasonable option when a tear is anatomically reparable. Comparative studies with control groups, objective functional and imaging endpoints, and longer follow-up are needed to confirm durability and any advantage in preventing osteoarthritis.
